# Role of chemotherapy in dedifferentiated liposarcoma of the retroperitoneum: defining the benefit and challenges of the standard

**DOI:** 10.1038/s41598-017-12132-w

**Published:** 2017-09-19

**Authors:** J. A. Livingston, D. Bugano, A. Barbo, H. Lin, J. E. Madewell, W. L. Wang, A. J. Lazar, W. W. Tseng, C. L. Roland, B. W. Feig, R. Pollock, A. P. Conley, R. S. Benjamin, S. Patel, N. Somaiah

**Affiliations:** 1 Department of Sarcoma Medical Oncology, The University of Texas MD Anderson Cancer Center, Houston, TX USA; 2 Department of Medical Oncology, Hospital Israelita Albert Einstein, São Paulo, Brazil; 3 Department of Biostatistics, The University of Texas MD Anderson Cancer Center, Houston, TX USA; 4 Department of Diagnostic Radiology, The University of Texas MD Anderson Cancer Center, Houston, TX USA; 5 Department of Pathology, The University of Texas MD Anderson Cancer Center, Houston, TX USA; 6 Department of Surgery, Section of Surgical Oncology, University of Southern California, Keck School of Medicine, Los Angeles, CA USA; 7 Department of Surgical Oncology, The University of Texas MD Anderson Cancer Center, Houston, TX USA

## Abstract

Benefit from chemotherapy for well-differentiated/de-differentiated (WD/DD) liposarcomas has been reported to be minimal, however traditional response criteria may not adequately capture positive treatment effect. In this study, we evaluate benefit from first-line chemotherapy and characterize imaging response characteristics in patients with retroperitoneal (RP) WD/DD liposarcoma treated at The University of Texas MD Anderson Cancer Center. Response was assessed using RECIST (Response Evaluation Criteria in Solid Tumors) and an exploratory analysis of vascular response was characterized. Among 82 patients evaluable for response to first-line therapy, 31 patients received neoadjuvant chemotherapy for localized/locally advanced disease; 51 received chemotherapy for unresectable recurrent/metastatic disease. Median overall survival from the start of chemotherapy was 29 months (95% CI 24–40 months). Response rates by RECIST: partial response (PR) 21% (17/82), stable disease (SD) 40%, and progression (PD) 39%. All RECIST responses were in patients receiving combination chemotherapy. A qualitative vascular response was seen in 24 patients (31%). Combination chemotherapy yields a response rate of 24% and a clinical benefit rate (CR/PR/SD > 6 months) of 44%, higher than previously reported in DD liposarcoma. A higher percentage of patients experience a vascular response with chemotherapy that is not adequately captured by RECIST in these large heterogeneous tumors.

## Introduction

Soft-tissue sarcomas (STS) represent a rare subset of malignancies, with liposarcomas being one of the most common histologic subtypes and the most common STS of the retroperitoneum (RP) among adults^1^. Well-differentiated/d edifferentiated (WD/DD) liposarcomas represent > 40% of liposarcomas accounting for around 1500 new cases per year and most commonly arise in the retroperitoneum (RP)^2^.

To date, surgery remains the mainstay of treatment for WD/DD liposarcomas of the RP, however local recurrence rates can be >80%^3^. Tumors are classified as DD liposarcoma when a part of the tumor has a high-grade typically non-lipogenic component along with a varying component of WD liposarcoma associated with it. DD liposarcoma is associated with a poorer prognosis. Benefit from chemotherapy for WD/DD liposarcomas has been reported to be minimal and limited to DD areas, with response rates reported ≤12%, and hence systemic therapy is not frequently utilized in the primary or recurrent setting^4,5^. Improved insight into the molecular characteristics of WD/DD liposarcoma has led to significant enthusiasm and recent trials of novel targeted therapies. However, response rates to MDM2 and CDK4 inhibitors have also been limited thus far, though prolonged stability has been reported^6,7^.

RECIST (Response Evaluation Criteria in Solid Tumors) is the most commonly used criteria for the assessment of treatment response in solid tumors, however previous reports have documented the limitations of RECIST in STS^8,9^. Retroperitoneal WD/DD liposarcoma tend to be large and heterogeneous, with varying components of high-grade and low-grade tumor often within the same mass. This makes response to chemotherapy challenging to assess by RECIST alone, since WD liposarcoma do not respond to chemotherapy and systemic chemotherapy is primarily used for the high-grade DD liposarcoma component. Due to these challenges, we felt it important to characterize response and evaluate clinical benefit of standard cytotoxic chemotherapy in order to serve as a baseline for future studies in DD liposarcoma. As dedifferentiated tumors are the more vascular/enhancing part of the tumor^10^, we hypothesized that assessing changes in vascularity in addition to RECIST might better identify responders. In this single-center retrospective study, we sought to evaluate benefit from first-line chemotherapy, explore changes in tumor imaging characteristics, and correlate these imaging findings with pathologic response in patients receiving neoadjuvant therapy.

## Results

### Patients

Between September 2002 to March 2014, we identified 330 patients with RP liposarcoma. Of the 330 patients, 84 received first or second line chemotherapy and had imaging available for response assessment. Two patients only had imaging available for response to second line therapy. The majority of patients had both WD and DD components in their tumor, but 17 patients exhibited predominantly dedifferentiated tumors at baseline by imaging characteristics, in which the entire visualized tumor demonstrated vascular enhancement. Table 1 summarizes clinicopathologic features.

**Table 1:** Patient characteristics.

Characteristics	Total N = 84 n (%)
Age at DD biopsy	
Mean (SD)	55.3 (10.78)
Median	56.5
Sex	
Female	35 (41.7)
Male	49 (58.3)
Race	
Caucasian	66 (78.6)
Hispanic	10 (11.9)
Asian	4 (4.8)
African-American	1 (1.2)
Pacific Islander	1 (1.2)
Neoadjuvant chemo	
No	53 (63.1)
Yes	31 (36.9)
Primary unresectable	
No	76 (90.5)
Yes	8 (9.5)
Combination agent	
No	10 (11.9)
Yes	74 (88.1)
Anthracycline	
No	17 (20.2)
Yes	67 (79.8)
Chemo regimen	
Other	41 (48.8)
A/I	43 (51.2)
Number of cycles during 1st line	
Median, Min - Max	4.0, 1.0–9.0
Receiving 2nd line	
No	43 (51.2)
Yes	41 (48.8)
Baseline imaging characteristics	
WD/DD by imaging	67 (79.8)
Predominantly DD by imaging	17 (20.2)

### Treatments

Thirty-one patients received neoadjuvant chemotherapy either for primary tumors or localized disease recurrences, 45 received chemotherapy for unresectable recurrent disease, and 8 received chemotherapy for primary unresectable disease. Front-line therapy consisted of combination chemotherapy in 74 cases (88%) and single agent therapy in 10 cases (12%). Sixty-seven (80%) patients received an anthracycline-containing regimen. Doxorubicin and ifosfamide (A/I) was the most common regimen as 1^st^ line therapy. The median number of chemotherapy cycles was 4 for all regimens (range 1–9). Forty-one patients received second-line therapy with a cytotoxic agent and 10 patients went on to receive a third-line cytotoxic chemotherapy. The most common regimen in the 2^nd^ line was combination gemcitabine and docetaxel (23/41, 56%) followed by doxorubicin and dacarbazine (7/41, 17%).

### Response

Eighty-two of 84 patients included in the study had imaging available to assess response to first-line chemotherapy. By RECIST 1.1, 17 patients (21%) had partial response, 33 patients (40%) had stable disease, and 32 (39%) had progressive disease. Response rates to neoadjuvant therapy were partial response 22% (7/31), stable disease 52% (16/31), and progressive disease 26% (8/31). Among 51 evaluable patients treated in the unresectable/metastatic setting, response rates were partial response 20% (10/51), stable disease 33% (17/51), and progressive disease 47% (24/51). There were no complete responses observed. The observed clinical benefit rate (CR, PR, or SD >6 months) was 38% (95% CI: 0.25–0.51) among all patients and was higher (44%, *p* = 0.04) in patients receiving combination chemotherapy. The objective response rate (ORR) for first-line chemotherapy was significantly higher in patients receiving combination therapy vs single agent therapy (24% vs 0%, *p* = 0.0019) and anthracycline containing regimens (26% vs 0%, *p* = 0.0011). Patients receiving doxorubicin and ifosfamide (A/I) had the highest response rate (13/43, 30%). RECIST response to first-line therapy by chemotherapy regimen is summarized in Table 2. Thirty-nine patients who received second-line chemotherapy were evaluable with partial response seen in 7 patients (18%), stable disease in 15 patients (38%), and progressive disease in 17 patients (44%). Responses were seen with combination gemcitabine/docetaxel, doxorubicin/dacarbazine, and bevacizumab/temozolamide. Gemcitabine/docetaxel had response rate of 17% (4/23) in the 2^nd^ line. None of the 4 patients with PR to gemcitabine/docetaxel had responses to first-line doxorubicin-containing combination regimens.

**Table 2:** RECIST response to first-line therapy by chemotherapy regimen.

	RECIST			
	Total N = 82 n (col %)	PD n (row %)	SD n (row %)	PRn (row %)
**Chemotherapy Regimen**				
Doxorubicin (A)	7 (8.5)	7 (100)	0 (0.0)	0 (0.0)
A + I	43 (51.2)	9 (21.4)	21 (48.8)	13 (30.2)
V + A + I	1 (1.2)	0 (0.0)	1 (100)	0 (0.0)
A + DTIC	12 (14.6)	3 (25)	6 (50)	3 (25)
Cy + A + DTIC	2 (2.4)	1 (50)	0 (0.0)	1 (0.0)
Gemcitabine (G)	2 (2.4)	1(50)	1 (50)	0 (0.0)
G + T	14 (18.3)	10 (71.4)	4 (28.6)	0 (0.0)
Other	1 (1.2)	1 (100)	0 (0.0)	0 (0.0)

Key: A = doxorubicin, I = ifosfamide, V = vincristine, DTIC = dacarbazine, Cy = cyclophosphamide, G = gemcitabine, T = docetaxel.

Seventy-seven patients had imaging (contrast-enhanced CT) evaluable for vascular response out of which a qualitative vascular imaging response was observed in 26 patients (34%) receiving front-line chemotherapy. Twenty-nine patients (38%) demonstrated stable vascularity and 22 patients (29%) demonstrated increased tumor vascularity following treatment. Eleven of 33 patients (33%) with stable disease by RECIST and 4 of 32 patients (13%) with progressive disease by RECIST had decreased vascularity. There was however a reasonable correlation between the 2 assessment methods (Cohen’s κ 0.26, 95% CI: 0.09–0.43, *p* = 0.001, Table 3).

**Table 3:** RECIST and vascular response assessment to first-line therapy.

	RECIST					
	Total N = 82 n (col %)	PD N = 32 (39%) n (row %)	SD N = 33 (40.2%) n (row %)	PR N = 17 (20.7%) n (row %)	Cohen’s Κ	*p* value
**Vascular response**						
PD	22 (26.8)	15 (68.2)	7 (31.8)	0 (0.0)	0.26 (0.09, 0.43)	0.001^1^
SD	29 (35.4)	12 (41.4)	13 (44.8)	4 (13.8)		
PR	26 (31.7)	4 (15.4)	11 (42.3)	11 (42.3)		
NA	5 (6.1)	1 (20.0)	2 (40.0)	2 (40.0)		

### Pathologic Response

**Figure 1:**
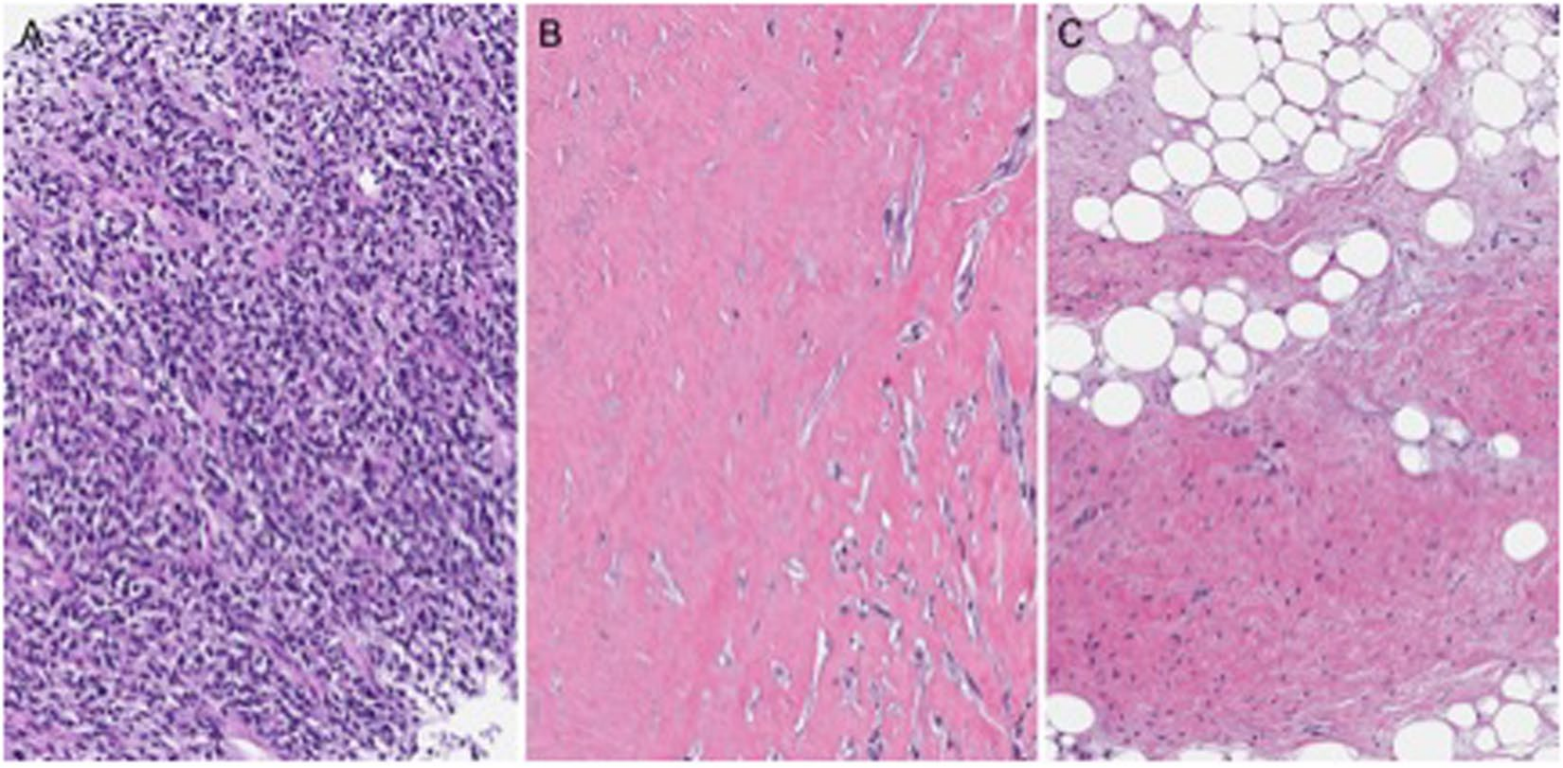
Pathologic changes in dedifferentiated liposarcoma following chemotherapy. (**A**) Pre-treatment biopsy. Dedifferentiated liposarcoma (H&E, 200x). (**B**) Post-treatment resection. Dedifferentiated liposarcoma with extensive treatment effect (decreased cellularity, hyalinization and necrosis (H&E, 200x). (**C**) Same post treatment resection adjacent well-differentiated component. Histological features of treatment are not seen in this component (H&E, 200x).

Twelve of 31 patients who received neoadjuvant chemotherapy had the resection specimens available for independent pathology review. All 12 tumors demonstrated variable pathologic response to treatment (median 60% overall treatment effect, range 5%-95%). Six patients had ≥70% treatment change in the dedifferentiated component Fig. 1. Only 1 of the 12 patients assessed had a response by RECIST (PR); 5 of 12 had qualitative vascular imaging responses with decreased vascularity.

### Progression-free and Overall Survival

The median follow-up was 38 months (95% CI: 26–61 months). Forty-six (55%) patients died and the median OS (from initiation of chemotherapy) was 29 months (95% CI: 24–40 months). Seven percent of the patients had died by 6 months, 19% by one year, and 40% by 2 years. Median OS from the first diagnosis of DD liposarcoma was 45 months (95% CI: 36–66 months).

Among patients receiving neoadjuvant chemotherapy, 24 (77%) had a recurrence and the median DFS was 10 months (95% CI: 4–23 months). Thirty-seven percent had recurred by 6 months, 52% by one year, and 71% by 2 years. Median OS (from initiation of chemotherapy) in this group was 33 months (95% CI: 22–NR) and median OS from the diagnosis of DD liposarcoma in this group was 41 months (95% CI: 30–NR).

**Figure 2:**
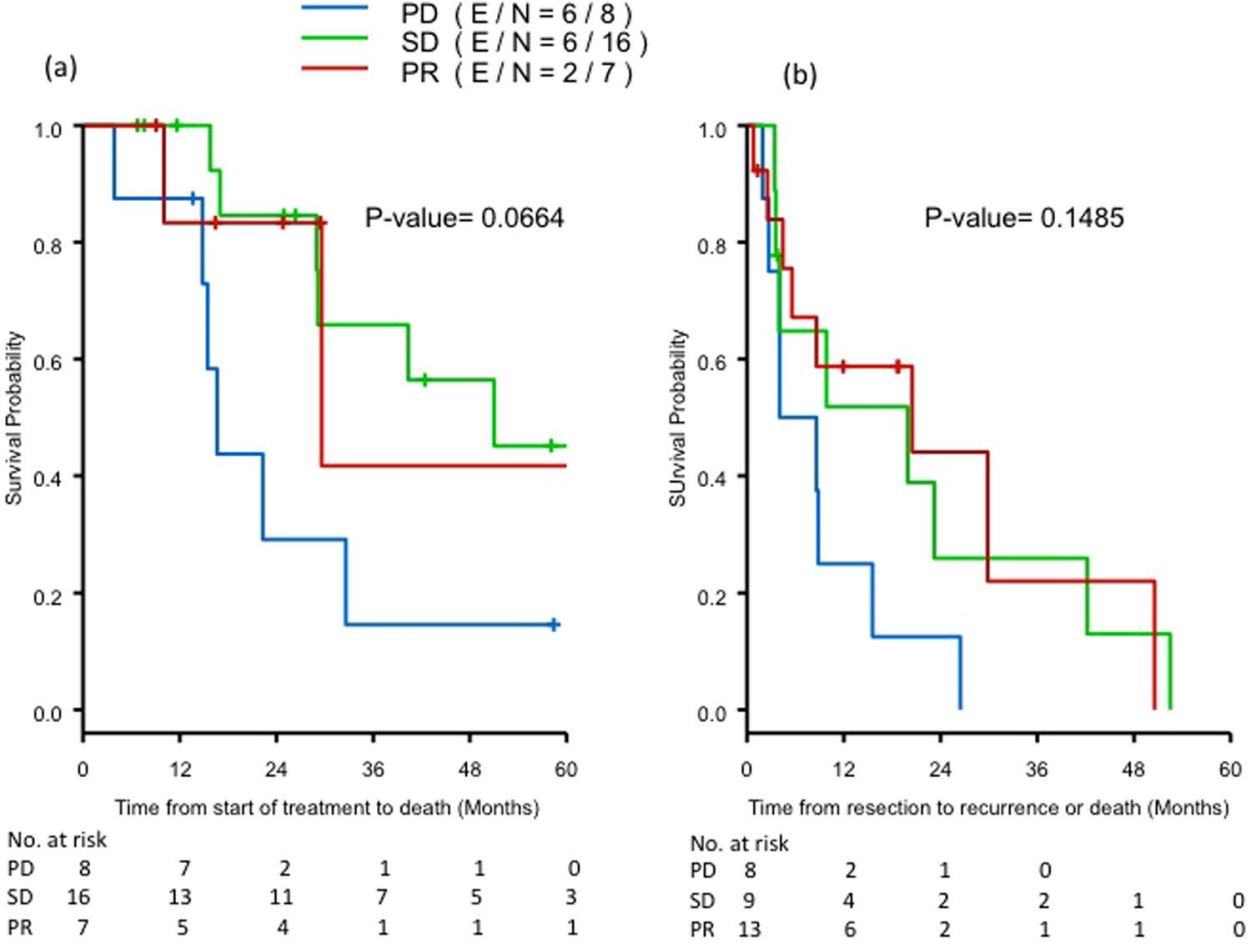
Survival for neoadjuvant patients. (2.1) Overall survival by RECIST (landmark analysis); (2.2) Disease-free survival by vascular response criteria.

Patients with PR or SD to neoadjuvant therapy had increased OS as compared to patients with PD (median OS 28 and 50 months vs 15 months, respectively), but this did not meet statistical significance, likely due to the small numbers of patients in the cohort (*p* = 0.06, Fig. 2a). Patients with increased vascularity following neoadjuvant therapy demonstrated a trend to shorter DFS as compared to those with stable or decreased vascularity (median 6.3 v 19.9 and 20.5 months, *p* = 0.15, Fig. 2b). When RECIST response and qualitative vascular response were analyzed together, neoadjuvant patients with PD by RECIST and PD by vascular criteria had the worst outcomes as compared to patients with PR/SD by RECIST and PR/SD by vascular criteria. Specifically, median DFS was 6.4 vs 19.9 months (*p* = 0.10) and median OS was 13.6 vs 49.5 months (*p* = 0.0017; Figs S1 and S2 respectively, online).

For patients receiving first-line therapy in the recurrent/metastatic setting, the median PFS was 4 months (95% CI: 3–7 months) with median OS (from initiation of chemotherapy) of 25 months (95% CI: 18–31 months).

### Prognostic Factors

Combination chemotherapy, age at diagnosis of dedifferentiated liposarcoma, and baseline tumor characteristics by imaging were associated with OS among all patients in univariate and multivariate analysis (all *p* < 0.05, Table S1.1 and S1.2). RECIST response status was associated with a trend toward improved OS among all patients (*p* = 0.087). Landmark analysis was performed and demonstrated the same associations with OS in univariate and multivariate models (Table S2.1 and S2.2). Patients who were older (age ≥mean age, 56.5y), had predominantly dedifferentiated tumors based upon baseline imaging or did not receive combination therapy had inferior OS.

Similarly in the patients receiving chemotherapy for unresectable or metastatic disease, age at diagnosis, baseline tumor characteristics by imaging, and combination chemotherapy were associated with OS (all *p* < 0*.05*, Table S3.1). In the multivariate model, predominantly dedifferentiated tumors by imaging had inferior OS (HR 2.71, 95% CI 1.22–6.00, Table S3.2). Combination therapy was associated with improved OS (HR 0.31, 95% CI 0.12–0.82).

## Discussion

This study represents the largest single-center experience with chemotherapy in retroperitoneal DD liposarcoma including the largest series of patients treated with neoadjuvant chemotherapy to date. The majority of new patients with WD/DD liposarcoma evaluated at MDACC during the study period underwent surgery as their primary therapy, with only 25% of patients receiving cytotoxic chemotherapy. Current clinical practice within our institution favors the use of chemotherapy in patients with DD liposarcoma who present with recurrence or in patients with primary DD liposarcoma if they are unresectable or borderline-resectable^11^. Patients with recurrent or borderline-resectable primary disease are evaluated for neoadjuvant combination chemotherapy. Other characteristics taken into consideration are performance status, comorbidities, extent of DD, and time to recurrence. In our study, the majority of patients received combination chemotherapy (88%), mainly anthracycline-containing regimens (80%). This is in significant contrast to other large multi-institutional series of WD/DD liposarcoma where the use of single-agent doxorubicin was more common and combination chemotherapy was given in approximately 40% of patients^4^. Further, the majority of patients in our center receive combination chemotherapy with anthracycline-based regimens,

The overall ORR of 21% within our study was significantly higher than previously reported in WD/DD liposarcoma. This may be due to a combination of factors; we excluded patients with exclusively WD histology and a higher proportion of patients received combination chemotherapy. There could also be variations in the regimens and the doses used across the studies. Combination chemotherapy is known to result in increased response rates in sarcoma patients^12^. In our study, objective responses by RECIST were only seen in patients receiving combination therapy. The ORR in patients receiving combination chemotherapy was 24% and in those receiving A/I was 30%. In the largest reported series of 208 patients across multiple institutions with advanced WD/DD liposarcoma by *Italiano et al*., the ORR was 12%, with no difference seen in ORR between WD and DD liposarcoma^4^. Similar to our study ORR was significantly higher for patients receiving combination anthracycline-based chemotherapy (18.5% vs 7.5% *p* = 0.04). Other studies have shown a higher response rate in DD liposarcoma as compared to WD liposarcoma, including a small retrospective series of 32 patients, which showed an ORR of 11% with all responses seen in DD liposarcoma^5^.

**Figure 3:**
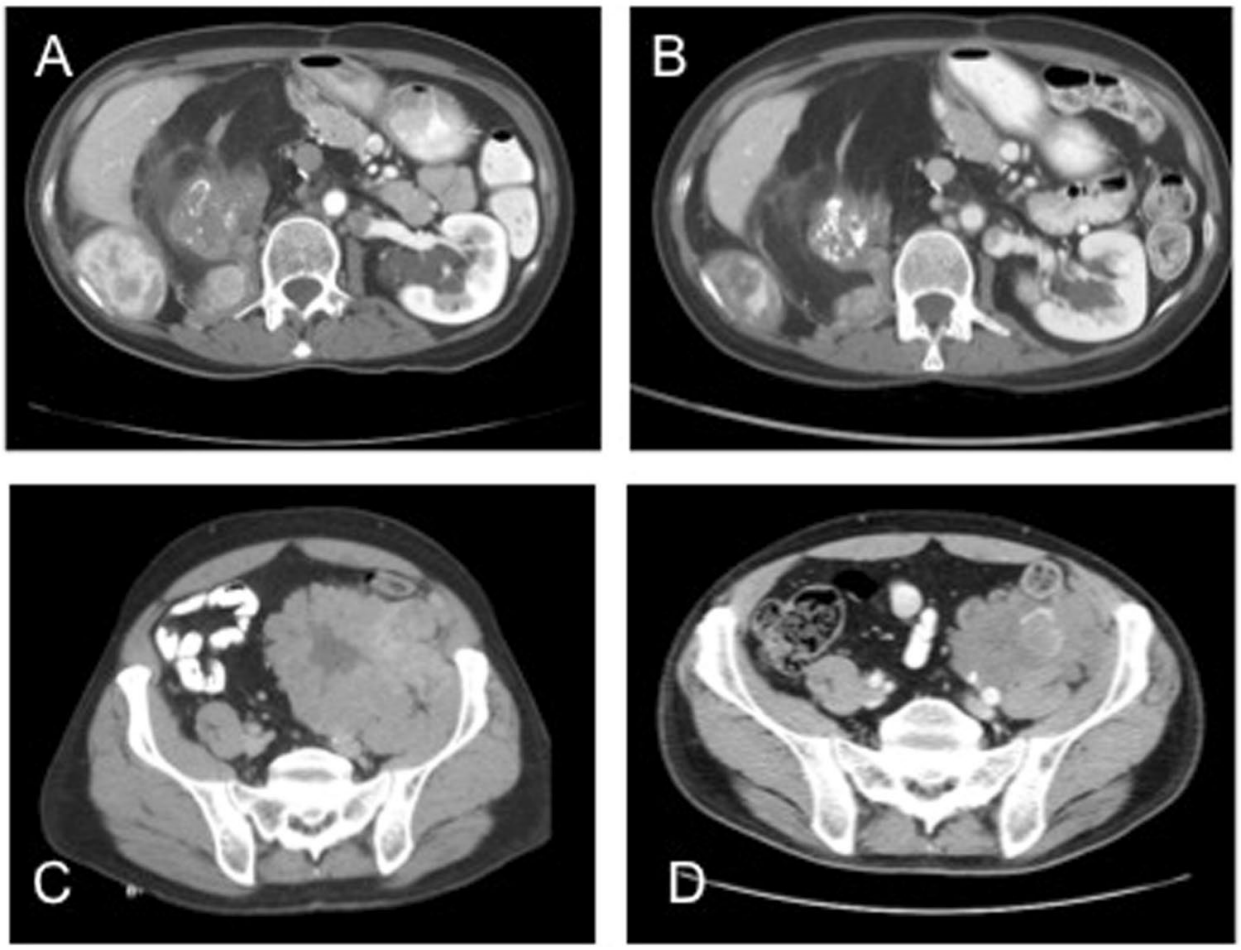
CT Characteristics of Response in WD/DD LPS. (**A**) Pre- and (**B**) Post-treatment with response features including decrease vascularity and increased calcification, and increase in fatty elements (WD/ALT); (**C**) Pre- and (**D**) Post-treatment with RECIST response and decreased vascularity with increased calcification.

Accurately evaluating response to treatment by RECIST is challenging because of the heterogeneity of WD/DD liposarcoma (Fig. S3). Given that the predominantly fatty portions of the tumor are unlikely to shrink, this might underestimate the response in the DD areas, which are the main areas of concern given the poorer prognosis of DD (Fig. 3). Within our series, 34% of patients demonstrated decreased tumor vascularity with treatment. Four patients with PD by RECIST demonstrated decreased vascularity and an additional 11 patients with SD had a response by our qualitative vascular criteria. Recently, our group has shown that positron emission tomography (PET/CT) can help identify areas of dedifferentiation accurately^13^ in WD/DD liposarcoma. PET/CT might not only help increase the accuracy of biopsy in identifying dedifferentiation but also might be more effective in determining response.

There are no standard criteria for determining a good pathologic response in soft tissue sarcomas, more specifically liposarcomas. For our study, we defined pathologic response and treatment effect broadly including necrosis, hyalinization, cytological changes, or any combination of these effects. Using these criteria, all 12 evaluable patients had evidence of some therapeutic effect on the tumor at resection following neoadjuvant chemotherapy. Six patients demonstrated >70% treatment effect, with 4 patients having ≥90% treatment effect in the dedifferentiated component. The role for pathologic response assessment in prognostication and modification of post-surgical therapy for dedifferentiated liposarcomas requires additional study.

The retrospective design of this study and the heterogeneity in the treatments received limited our ability to assess survival benefit based upon response. In the unresectable/metastatic setting, OS was not significantly different based upon response to first-line therapy by either RECIST or vascular changes and this could be due to the limited number of patients and confounding factors. Some patients went on to receive debulking surgeries and there was variability in subsequent lines of therapy, which may have impacted OS. In the neoadjuvant setting, combination therapy was associated with improved DFS, and improved OS, which could represent a selection bias as sicker patients with poor PS and/or significant comorbidities may not have been offered treatment with combination chemotherapy.

Our study shows that DD liposarcomas respond to chemotherapy and though response rates are not as high as for myxoid liposarcoma, their chemosensitivity is not very different when compared to the benchmark for other soft tissue sarcomas^12^. Patients should be considered for combination chemotherapy when appropriate. Traditional response criteria might underestimate the response in these generally large heterogeneous tumors and hence expert interpretation of imaging is important.

Standard chemotherapy is effective and yields higher response rates than previously reported in WD/DD liposarcoma of the retroperitoneum. Combination chemotherapy should be considered in DD liposarcoma when tumor shrinkage is critical, especially in those patients with borderline-resectable tumors. This study points to the difficulty and potential underestimation of response using standard RECIST criteria for WD/DD liposarcoma and begs the need for additional studies to prospectively test better strategies (PET/CT or vascular response) for response assessment and to determine long-term benefit from chemotherapy in this population.

## Patients and Methods

### Patients

A retrospective review of the University of Texas MD Anderson Cancer Center pathology database was undertaken to identify patients with RP WD/DD liposarcoma treated within our center from 9/2002 to 3/2014. Inclusion criteria were age ≥ 18 years, histologically proven DD liposarcoma of the RP, treatment with systemic chemotherapy, and CT imaging available for response assessment. Of note, the clinical practice within our center is to recommend chemotherapy only in patients who have histologically confirmed DD along with imaging characteristics to support dedifferentiation. First-line therapy was defined as the first systemic therapy received, either neoadjuvant or in the unresectable/metastatic setting. In all cases, the diagnosis was established according to the World Health Organization Classification of Tumors by an expert sarcoma pathologist^14^. Patients with exclusively WD liposarcoma, those who did not receive treatment within our center, or those who did not have baseline and post-treatment imaging available for review were excluded. Institutional Review Board approval was obtained for this retrospective chart review and was exempt from requiring informed consent.

### Treatment

Patients were treated at the discretion of their primary physician in collaboration with a multi-disciplinary tumor board using regimens that were standard at the time of treatment or according to ongoing institutional/multi-institutional clinical studies.

### Response Assessment

CT scans were obtained originally with a multi-channel, multi-detector CT scanner using 5-mm or 2.5-mm slice thickness. Examinations were performed with intravenous contrast injection of 125 to 150 mL of contrast media (Omnipaque 350, Nycomed Amersham; later Optiray 350, Mallinckrodt) at a rate of 3 mL per second. An abdominal post-contrast CT was obtained with an approximate delay of 60 seconds. Patients received oral barium sulfate suspension (900 mL) for bowel contrast and rectal barium sulfate suspension as needed. For the purpose of this study, a board-certified radiologist with specialized expertise in soft tissue tumors (JEM) who was blinded to clinical outcomes reviewed pre- and post-treatment CT scans, initially to assess changes in tumor vascularity (defined below). The best response to treatment was also evaluated according to RECIST 1.1.^15^ RECIST measurements/assessment was initially completed by a fellow (DB) and later reviewed by the radiologist (JEM) after completion of and separated in time (by greater than 5 months) from the vascular response assessment. Of note, in cases where the DD liposarcoma component was distinctly demarcated, the response measurements were focused on the DD component. Axial and reformatted images were reviewed on a PACS workstation (iSite; Stentor Inc., Brisbane, CA) with soft tissue window/level (500 window width/55 window level). All time points of imaging while on treatment were recorded and analyzed. Qualitative changes in tumor vascularity were evaluated by 2 imaging metrics: vascular tumor volume and intensity (contrast enhancement). Progressive disease (PD) by vascular assessment was defined as increase in vascular tumor volume or intensity of vascularity as compared to baseline; stable disease (SD) was defined as no significant change in either vascular tumor volume or intensity; partial response (PR) was defined as decrease in vascular tumor volume or intensity of vascularity as compared to baseline. Patients who had non-contrasted studies were classified as not applicable (NA). These metrics were independent of overall tumor volume.

### Pathologic Response

An expert sarcoma pathologist independently reviewed all available slides from pre-treatment biopsy and resection specimens from patients receiving neoadjuvant chemotherapy. Histologic features of treatment effect include decreased cellularity, necrosis, hyalinization, cytological changes (degenerative bizarre nuclei), or any combination of these effects. Treatment response was assessed as a percentage of tumor which exhibited histologic features of treatment effect. No treatment effect was observed in any well-differentiated component and was seen only in the dedifferentiated component.

### Statistical Analysis

Baseline characteristics were summarized using descriptive statistics^16^. Cohen’s kappa statistic, κ, was computed to assess the agreement between categorical RECIST and vascular responses^17^. Chi-square test was used to determine association between baseline vascularity and RECIST while Fisher’s Exact test was used to compare RECIST/vascular response and path response among neoadjuvant patients. Overall survival (OS, time from 1st chemo until death), disease-free survival (DFS) among neoadjuvant patients (time from resection until recurrence or death), and progression-free survival (PFS) among 1st line unresectable patients (time from 1st chemo until progression or death) were estimated by the Kaplan-Meier method^18^. The clinical benefit rate was defined as the rate of complete or partial response or stable disease of at least 6 months duration by RECIST. Log-rank test^19^ was performed to test the difference in survival between groups. Regression analysis using Cox proportional hazards model^20^ was conducted for all baseline characteristics, and for the RECIST and vascular assessment methods. A landmark analysis was performed in which the time between start of chemo and initial response assessment (~1.5 months) was subtracted from the OS and PFS times. One patient who progressed less than 1.5 months after 1st chemo was excluded in the landmark analysis only. Statistical significance was determined using a two-sided p-value <0.05. All statistical analyses were done using SAS 9.3 (SAS Institute, Inc., Cary, NC).

## Electronic supplementary material

Supplementary Tables and Figures
