# Supplementary material for: Role of chemotherapy in dedifferentiated liposarcoma of the retroperitoneum: defining the benefit and challenges of the standard
Source: Sci Rep. 2017 Sep 19;7:11836. doi: 10.1038/s41598-017-12132-w (PMC5605500; doi:10.1038/s41598-017-12132-w)
Supplement: Supplementary file 1 — Supplementary Tables and Figures [file 41598_2017_12132_MOESM1_ESM.pdf]

Supplemental Tables and Figures

**Role of chemotherapy in dedifferentiated liposarcoma of the retroperitoneum: defining the benefit and challenges of the standard.**

J. A. Livingston MD[1], D. Bugano MD[2], A. Barbo[3], H. Lin PhD[3], J. E Madewell MD[4], W. Wang MD[5], A. Lazar MD PhD[5], W. Tseng MD[6], C. L. Roland MD[7], B. Feig MD[7], R. Pollock MD[7], A. P. Conley MD[1], R. S. Benjamin MD[1], S. Patel MD[1], and N. Somaiah MD[1*]

**Supplemental Tables and Figures (Online Only).**

**Supplemental Table S1.1 Univariate hazard models - all patients and neoadjuvant patients**

| | All patients | | | | Neoadjuvant patients | | | | | | | |
|---|---|---|---|---|---|---|---|---|---|---|---|---|
| | Overall survival | | | | Overall survival | | | | Disease-free survival | | | |
| Parameter | Estimate (s.e.) | p value | pVal for overall effects | Hazard ratio (95% CI) | Estimate (s.e.) | p value | pVal for overall effects | Hazard ratio (95% CI) | Estimate (s.e.) | p value | pVal for overall effects | Hazard ratio (95% CI) |
| **RECIST** | | | | | | | | | | | | |
| PD | NA | | | | NA | | | | -0.03 (0.65) | 0.959 | 0.978 | 0.97 (0.27, 3.49) |
| SD | NA | | | | NA | | | | 0.07 (0.58) | 0.909 | | 1.07 (0.34, 3.32) |
| PR | NA | | | | NA | | | | REF | | | |
| **Vascular response assessment** | | | | | | | | | | | | |
| PD | NA | | | | NA | | | | 0.90 (0.52) | 0.083 | 0.170 | 2.45 (0.89, 6.72) |
| SD | NA | | | | NA | | | | 0.06 (0.52) | 0.910 | | 1.06 (0.38, 2.96) |
| PR | NA | | | | NA | | | | REF | | | |
| **Age at DD biopsy** | | | | | | | | | | | | |
| <56.5 years | REF | | | | REF | | | | REF | | | |
| >=56.5 years | 0.82 (0.31) | 0.007 | | 2.28 (1.25, 4.15) | 0.82 (0.56) | 0.143 | | 2.28 (0.76, 6.87) | 0.47 (0.43) | 0.271 | | 1.61 (0.69, 3.73) |
| **Sex** | | | | | | | | | | | | |
| Female | REF | | | | REF | | | | REF | | | |
| Male | 0.24 (0.31) | 0.433 | | 1.28 (0.69, 2.34) | 1.07 (0.76) | 0.161 | | 2.92 (0.65, 13.08) | -0.02 (0.49) | 0.963 | | 0.98 (0.37, 2.57) |
| **Race** | | | | | | | | | | | | |
| Caucasian | REF | | 0.663 | | REF | | 0.999 | | REF | | 0.734 | |
| Hispanic | -0.20 (0.48) | 0.672 | | 0.82 (0.32, 2.08) | -0.04 (0.77) | 0.958 | | 0.96 (0.21, 4.31) | 0.16 (0.64) | 0.806 | | 1.17 (0.34, 4.06) |
| Other | -0.60 (0.73) | 0.406 | | 0.55 (0.13, 2.27) | -16.22 (2403.54) | 0.995 | | 0.00 (0.00, ) | -0.74 (1.04) | 0.473 | | 0.48 (0.06, 3.62) |
| **Baseline imaging characteristics** | | | | | | | | | | | | |
| WD/DD | REF | | | | REF | | | | REF | | | |
| Predominantly DD | 0.87 (0.40) | 0.028 | | 2.39 (1.10, 5.20) | 0.35 (0.80) | 0.657 | | 1.43 (0.30, 6.83) | 0.26 (0.65) | 0.682 | | 1.30 (0.37, 4.62) |
| **Chemo regimen** | | | | | | | | | | | | |
| A/I | -0.41 (0.30) | 0.163 | | 0.66 (0.37, 1.18) | -0.98 (0.83) | 0.239 | | 0.38 (0.07, 1.91) | 0.23 (0.76) | 0.760 | | 1.26 (0.29, 5.56) |

| | All patients | | | | Neoadjuvant patients | | | | | | | |
|---|---|---|---|---|---|---|---|---|---|---|---|---|
| | Overall survival | | | | Overall survival | | | | Disease-free survival | | | |
| Parameter | Estimate (s.e.) | p value | pVal for overall effects | Hazard ratio (95% CI) | Estimate (s.e.) | p value | pVal for overall effects | Hazard ratio (95% CI) | Estimate (s.e.) | p value | pVal for overall effects | Hazard ratio (95% CI) |
| Other | REF | | | | REF | | | | REF | | | |
| **Combination chemotherapy** | | | | | | | | | | | | |
| No | REF | | | | NA | | | | NA | | | |
| Yes | -1.34 (0.47) | 0.004 | | 0.26 (0.10, 0.65) | NA | | | | NA | | | |
| **Anthracycline** | | | | | | | | | | | | |
| No | REF | | | | REF | | | | REF | | | |
| Yes | -0.23 (0.37) | 0.531 | | 0.79 (0.38, 1.64) | -31.00 (984026) | 1.000 | | 0.00 (0.00, ) | -3.37 (1.41) | 0.017 | | 0.03 (0.00, 0.55) |

**Supplemental Table S1.2 Multivariate hazard model for death among all patients**

| Parameter | Estimate (s.e.) | p value | Hazard ratio (95% CI) |
|---|---|---|---|
| **Age at DD biopsy** | | | |
| <56.5 years | REF | | |
| >=56.5 years | 0.68 (0.32) | 0.035 | 1.97 (1.05, 3.70) |
| **Baseline imaging characteristics** | | | |
| WD/DD | REF | | |
| Predominantly DD | 1.00 (0.41) | 0.014 | 2.71 (1.22, 6.00) |
| **Combination agent** | | | |
| No | REF | | |
| Yes | -1.17 (0.50) | 0.018 | 0.31 (0.12, 0.82) |

**Supplemental Table S2.1 Univariate hazard model for death among all patients and neoadjuvant patients - a landmark analysis**

| | All patients | | | | Neoadjuvant patients | | | |
|---|---|---|---|---|---|---|---|---|
| Parameter | Estimate (s.e.) | p value | pVal for overall effects | Hazard ratio (95% CI) | Estimate (s.e.) | p value | p value for overall effects | Hazard ratio (95% CI) |
| **RECIST** | | | | | | | | |
| PD | 0.79 (0.44) | 0.073 | 0.087 | 2.21 (0.93, 5.27) | 1.14 (0.82) | 0.163 | 0.089 | 3.14 (0.63, 15.63) |
| SD | 0.17 (0.45) | 0.696 | | 1.19 (0.50, 2.86) | -0.08 (0.82) | 0.927 | | 0.93 (0.19, 4.64) |
| PR | REF | | | | REF | | | |
| **Vascular response assessment** | | | | | | | | |
| PD | 0.15 (0.38) | 0.689 | 0.878 | 1.16 (0.55, 2.45) | 0.60 (0.68) | 0.375 | 0.663 | 1.83 (0.48, 6.95) |
| SD | -0.03 (0.37) | 0.927 | | 0.97 (0.47, 2.01) | 0.23 (0.68) | 0.735 | | 1.26 (0.33, 4.75) |
| PR | REF | | | | REF | | | |
| **Age at DD biopsy** | | | | | | | | |
| <56.5 years | REF | | | | REF | | | |
| >=56.5 years | 0.82 (0.31) | 0.007 | | 2.28 (1.25, 4.15) | 0.82 (0.56) | 0.143 | | 2.28 (0.76, 6.87) |
| **Sex** | | | | | | | | |
| Female | REF | | | | REF | | | |
| Male | 0.24 (0.31) | 0.433 | | 1.28 (0.69, 2.34) | 1.07 (0.76) | 0.161 | | 2.92 (0.65, 13.08) |
| **Race** | | | | | | | | |
| Caucasian | REF | | 0.663 | | REF | | 0.999 | |
| Hispanic | -0.20 (0.48) | 0.672 | | 0.82 (0.32, 2.08) | -0.04 (0.77) | 0.958 | | 0.96 (0.21, 4.31) |
| Other | -0.60 (0.73) | 0.406 | | 0.55 (0.13, 2.27) | -16.22 (2403.54) | 0.995 | | 0.00 (0.00, ) |
| **Baseline imaging characteristics** | | | | | | | | |
| WD/DD | REF | | | | REF | | | |
| Predominantly DD | 0.87 (0.40) | 0.028 | | 2.39 (1.10, 5.20) | 0.35 (0.80) | 0.657 | | 1.43 (0.30, 6.83) |
| **Chemo regimen** | | | | | | | | |
| A/I | -0.41 (0.30) | 0.163 | | 0.66 (0.37, 1.18) | -0.98 (0.83) | 0.239 | | 0.38 (0.07, 1.91) |
| Other | REF | | | | REF | | | |
| **Combination agent** | | | | | | | | |
| No | REF | | | | NA | | | |
| Yes | -1.34 (0.47) | 0.004 | | 0.26 (0.10, 0.65) | NA | | | |
| **Anthracycline** | | | | | | | | |
| No | REF | | | | REF | | | |
| Yes | -0.23 (0.37) | 0.531 | | 0.79 (0.38, 1.64) | -31.00 (984026) | 1.000 | | 0.00 (0.00, ) |

**Supplemental Table S2.2 Multivariate hazard model for death among all patients - a landmark analysis**

| Parameter | Estimate (s.e.) | p value | Hazard ratio (95% CI) |
|---|---|---|---|
| **RECIST** | | | |
| PD | 1.24 (0.46) | 0.007 | 3.45 (1.40, 8.53) |
| SD | 0.44 (0.45) | 0.323 | 1.56 (0.65, 3.76) |
| PR | REF | | |
| **Age at DD biopsy** | | | |
| <56.5 years | REF | | |
| >=56.5 years | 1.06 (0.33) | 0.001 | 2.90 (1.52, 5.53) |
| **Baseline imaging characteristics** | | | |
| WD/DD | REF | | |
| Predominantly DD | 1.03 (0.41) | 0.011 | 2.81 (1.27, 6.25) |

**Supplemental Table S3.1 Univariate hazard models recurrent/metastatic patients**

| | Overall survival | | | | Progression-free survival | | | |
|---|---|---|---|---|---|---|---|---|
| Parameter | Estimate (s.e.) | p value | pVal for overall effects | Hazard ratio (95% CI) | Estimate (s.e.) | p value | pVal for overall effects | Hazard ratio (95% CI) |
| **Age at DD biopsy** | | | | | | | | |
| <56.5 years | REF | | | | REF | | | |
| >=56.5 years | 0.92 (0.37) | 0.013 | | 2.50 (1.21, 5.18) | 0.49 (0.30) | 0.103 | | 1.63 (0.91, 2.93) |
| **Sex** | | | | | | | | |
| Female | REF | | | | REF | | | |
| Male | 0.11 (0.36) | 0.752 | | 1.12 (0.56, 2.26) | -0.13 (0.28) | 0.653 | | 0.88 (0.51, 1.53) |
| **Race** | | | | | | | | |
| Caucasian | REF | | 0.524 | | REF | | 0.761 | |
| Hispanic | -0.40 (0.61) | 0.515 | | 0.67 (0.20, 2.23) | -0.14 (0.44) | 0.748 | | 0.87 (0.36, 2.06) |
| Other | 0.65 (0.75) | 0.382 | | 1.92 (0.44, 8.31) | 0.38 (0.61) | 0.531 | | 1.46 (0.44, 4.82) |
| **Baseline imaging characteristics** | | | | | | | | |
| WD/DD | REF | | | | REF | | | |
| Predominantly DD | 1.16 (0.46) | 0.013 | | 3.17 (1.28, 7.89) | 0.82 (0.37) | 0.028 | | 2.26 (1.09, 4.69) |
| **Chemo regimen** | | | | | | | | |
| A/I | 0.13 (0.37) | 0.722 | | 1.14 (0.55, 2.38) | -0.09 (0.30) | 0.764 | | 0.91 (0.51, 1.65) |
| Other | REF | | | | REF | | | |
| **Combination agent** | | | | | | | | |
| No | REF | | | | | | | |
| Yes | -1.06 (0.49) | 0.030 | | 0.35 (0.13, 0.90) | | | | |
| **Anthracycline** | | | | | | | | |
| No | REF | | | | REF | | | |
| Yes | 0.25 (0.41) | 0.541 | | 1.29 (0.57, 2.87) | -0.47 (0.32) | 0.138 | | 0.63 (0.34, 1.16) |

**Supplemental Table S3.2 Multivariate hazard model for death among recurrent/metastatic patients**

| Parameter | Estimate (s.e.) | p value | Hazard ratio (95% CI) |
|---|---|---|---|
| **Age at DD biopsy** | | | |
| <56.5 years | REF | | |
| >=56.5 years | 0.68 (0.32) | 0.035 | 1.97 (1.05, 3.70) |
| **Baseline imaging characteristics** | | | |
| WD/DD | REF | | |
| Predominantly DD | 1.00 (0.41) | 0.014 | 2.71 (1.22, 6.00) |
| **Combination agent** | | | |
| No | REF | | |
| Yes | -1.17 (0.50) | 0.018 | 0.31 (0.12, 0.82) |

## Supplemental Figures

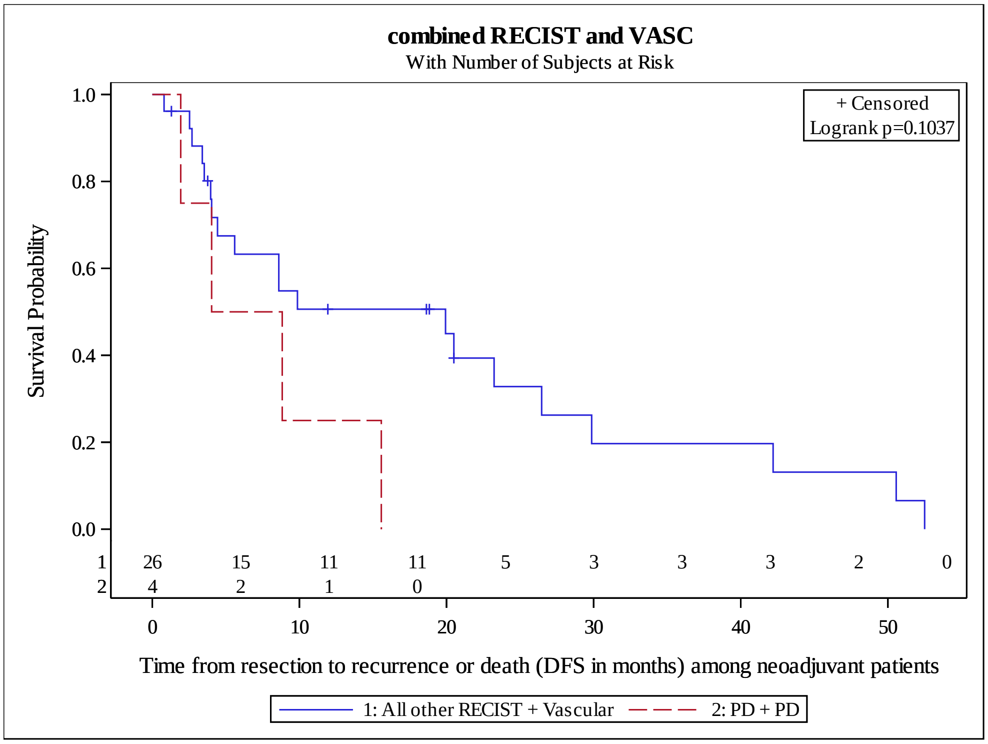


**Supplemental Figure S1. Disease-free survival amongst neoadjuvant patients by combined RECIST and vascular response criteria.**

Disease-free survival among neoadjuvant patients with progressive disease (PD) by both RECIST and vascular response criteria vs patients with partial response (PR) or stable disease (SD)

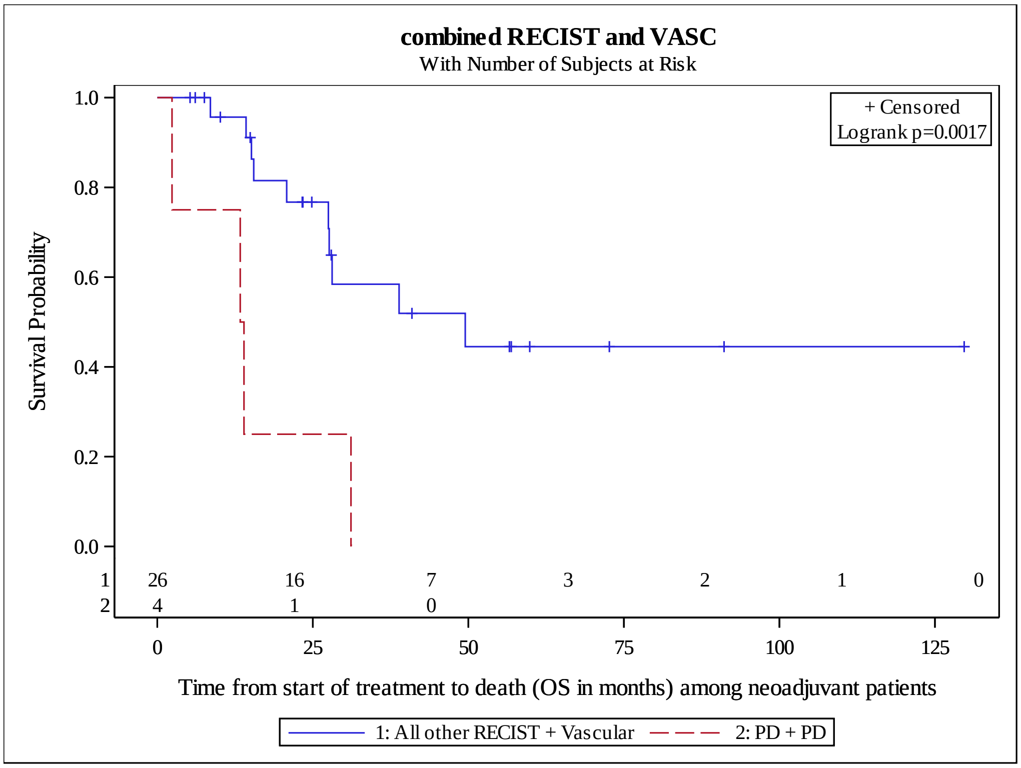


**Supplemental Figure S2. Overall Survival amongst neoadjuvant patients by combined RECIST and vascular response criteria.**

Overall survival among neoadjuvant patients with progressive disease (PD) by both RECIST and vascular response criteria vs patients with partial response (PR) or stable disease (SD)

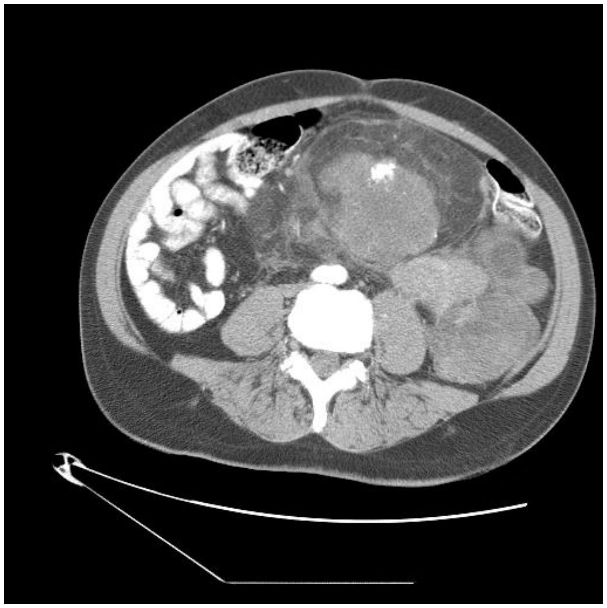

**Supplemental Figure S3. Heterogeneity of imaging characteristics in WD/DD liposarcoma.**

Baseline CT of WD/DD liposarcoma demonstrates highly vascularized portions, fatty components, and calcification.
